# Epstein–Barr virus detection in endoscopic submucosal dissection‐proven early gastric cancer with mixed‐type histology

**DOI:** 10.1002/cnr2.1730

**Published:** 2022-11-24

**Authors:** Hideo Yanai, Daisuke Chihara, Megumi Harano, Eiki Sakaguchi, Seiji Kaino, Tomoyuki Murakami, Jun Nishikawa

**Affiliations:** ^1^ Department of Clinical Research National Hospital Organization Kanmon Medical Canter Shimonoseki Japan; ^2^ Department of Gastroenterology & Hepatology Narional Hospital Organization Kanmon Medical Center Shimonoseki Japan; ^3^ Department of Pathology National Hospital Organization Kanmon Medical Center Shimonoseki Japan; ^4^ Department of Laboratory Science Yamaguchi University Graduate School of Medicine Ube Japan

**Keywords:** endoscopic submucosal dissection, Epstein–Barr virus‐associated gastric cancer, gastric cancer with mixed‐type histology

## Abstract

**Backgrounds:**

Early gastric cancer (EGC) with mixed‐type histology is a significant risk factor for additional surgery after endoscopic submucosal dissection (ESD). On the other hand, Epstein–Barr virus‐associated gastric cancer (EBVaGC) with mixed‐type histology is a favorable risk factor with regard to lymph node metastasis.

**Methods:**

We performed EBV detection in 13 ESD‐proven lesions in 13 cases of early gastric cancer with mixed‐type histology using EBV‐encoded small RNA1 in situ hybridization (EBER1 ISH).

**Results:**

EBVaGC was diagnosed in only one (7.7%) of the tested lesions. That EBVaGC patient underwent surgery and there was no residual lesion and no lymph metastasis. EBVaGC is not frequent in EGC with mixed‐type histology.

**Conclusions:**

EBV testing of gastric biopsy specimens seems not to be useful to predict the mixed‐type histology results of ESD. However, EBV testing for ESD specimens of EGC with mixed‐type histology is expected to be useful for avoiding excessive additional surgery.

## 1 INTRODUCTION

Recently, endoscopic submucosal dissection (ESD) is widely accepted as a first‐line treatment modality for early gastric cancer (EGC) with a low risk of lymph node (LN) metastasis. EGC lesions of clinically diagnosed differentiated type adenocarcinoma limited to within the mucosal layer are an absolute indication for ESD.^1^, ^2^, ^3^

However, we encountered 13 ESD‐proven EGC lesions with mixed‐type histology of differentiated and undifferentiated adenocarcinoma among 318 consecutive ESD lesions examined in our hospital. EGC lesions with mixed‐type histology are known as a significant risk factor for additional surgery after endoscopic submucosal dissection. Actually, 11 of the 13 lesions were indicated for additional surgery. Twelve of the gastric biopsy specimens were classified into Group 5 (differentiated type) and one into Group 4. Therefore, the mixed‐type histology of all 13 lesions was not expected in pretreatment clinical diagnosis. These EGC lesions with mixed‐type histology present a problem with regard to the ESD indication in the clinical diagnosis of EGC.^4^, ^5^

On the other hand, early Epstein–Barr virus‐associated gastric cancer (EBVaGC) is known to be a favorable risk factor with regard to LN metastasis.^6^ Early EBVaGC is expected to be within the extended criteria of the ESD indication.

EBV is a ubiquitous human herpes virus discovered in a Burkitt lymphoma cell. EBV is associated with a variety of tumors derived from B cells, such as Burkitt lymphoma, post‐transplant lymphoproliferative disease, and Hodgkin's disease. EBV is also detected in epithelial tumors, including nasopharyngeal carcinoma and GC.^7^ All GC cells of one EVCaGC lesion have monoclonal EBV plasmids, and a causal role of EBV in carcinogenesis is suspected.^8^

EBVaGC accounts for almost 9% of GC and consists of mainly lymphocyte‐rich undifferentiated‐type adenocarcinoma (carcinoma with lymphoid stroma, CLS) and some differentiated‐type adenocarcinoma. This means that EBVaGC has a mixed‐type histology.^9^, ^10^, ^11^, ^12^

Pretherapeutic EBV detection may be useful for prediction of EGC lesions with mixed‐type histology. Considering these backgrounds, we performed EBV detection in ESD‐proven EGC lesions with mixed‐type histology.

## 2 METHODS

We examined 13 lesions of 13 cases of ESD‐proven EGC with mixed‐type histology among 318 consecutive ESD‐treated EGC lesions from April 2004 through March 2012 in our hospital. The patients consisted of 11 males and 2 females, aged 53 to 86 years. Details of the cases are presented in Table 1. In EBVaGC lesions, EBV‐encoded small RNA1 (EBER1) is detected in all cancer cell nuclei by using EBER1 in situ hybridization (ISH).^13^ EBER1 is detected with a digoxigenin‐labeled 30‐base oligomer. In the present study, EBV was tested for using EBER1 ISH in situ hybridization for the ESD specimens.

**TABLE 1:** Thirteen cases of endoscopic submucosal dissection (ESD)‐proven gastric cancer with mixed‐type histology

Sex	Age	Macroscopic type	Location	Biopsy		Invasion depth	Curative result of ESD	Additional surgical operation: Result
**EBV‐associated gastric cancer**								
Male	73	0I	Remnant stomach	Group 5	(tub2)	pT1b (SM2)	No	Operated: No residual tumor
**EBV‐negative gastric cancer**								
Male	77	0IIc	Gastric body	Group 5	(tub2)	pT1a (M)	No	None (Rejected)
Male	59	0IIa	Gastric body	Group 5	(tub1)	pT1a (M)	No	Operated: Local residue
Male	70	0IIa + IIc	Cardiac area	Group 5	(tub2)	pT1b (SM2)	No	Operated: Intramural metastasis
Male	53	0IIa + IIc	Gastric body	Group 5	(tub2)	pT1b (SM2)	No	Operated: No residual tumor
Male	67	0I + IIa	Gastric body	Group 5	(tub1)	pT1b (SM2)	No	Operated: No residual tumor
Male	80	0IIc	Antral area	Group 5	(tub2)	pT1a (M)	No	None (Rejected)
Female	79	0I	Gastric body	Group 5	(tub2)	over pT1b (over SM2)	No	Operated: Subserosal invasion with LN metastasis
Male	68	0IIc	Gastric body	Group 5	(tub1)	pT1b (SM2)	No	Operated: LN metastasis
Male	86	0IIc	Gastric body	Group 5	(tub1)	pT1b (SM1)	No	Operated: LN metastasis
Male	64	0IIc	Antral area	Group 5	(tub1)	pT1a (M)	Yes	None
Female	60	0I	Cardiac area	Group 4		pT1a (M)	Yes	None
Male	76	0IIc	Gastric body	Group 5	(tub2)	pT1a (M)	No	Operated: Local residue

*Note*: Age, years old; pT1a (M), tumor limited to within mucosal layer; pT1b (SM1), tumor invading into submucosal layer within 500 μm; pT1b (SM2), tumor invading into submucosal layer over 500 μm; LN, lymph node. Additional D2 operation clarified residual lesions and LN metastasis. Well‐differentiated tubular adenocarcinoma (tub1) moderately differentiated tubular adenocarcinoma (tub2).

## 3 RESULTS

Of the 13 tested lesions, only one (7.7%) was EBER‐1‐positive EBVaGC (Table 1).

**FIGURE 1:**
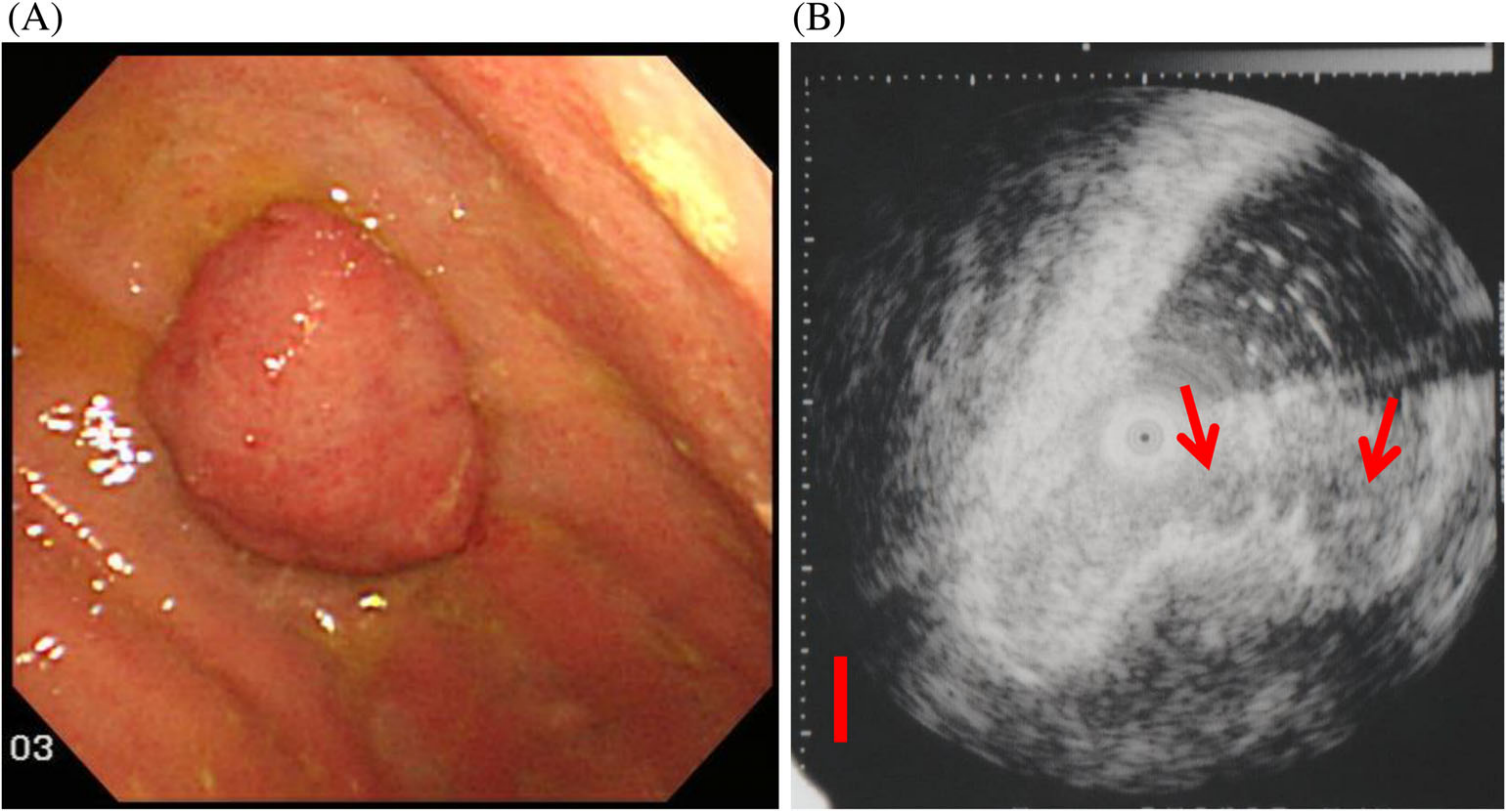
Endoscopic features of the ESD‐proven early gastric cancer lesion with differentiated and undifferentiated adenocarcinoma mixed‐type histology. The lesion was confirmed to be EBVaGC. The patient was a 73‐year‐old male. He had undergone partial gastrectomy and treatment of liver cancer. Gastric biopsy from his remnant stomach revealed second gastric cancer. The gastric biopsy specimen was classified as moderately differentiated adenocarcinoma, Group 5 (tub2). Less‐invasive ESD was chosen as the first‐line treatment for his cancer of the remnant stomach. (A) Endoscopic features. The EGC lesion is the protruding type (0‐I), almost three centimeters in diameter and located on the greater curvature of the remnant stomach. Endoscopy indicates an ordinary differentiated type EGC lesion within the mucosal layer. (B) Endosonographic features. There is irregular narrowing of the third hyperechoic layer, which corresponds to the submucosal layer (arrow). Tumor invasion into the submucosal layer was suspected. Scale bar: 5 mm.

**FIGURE 2:**
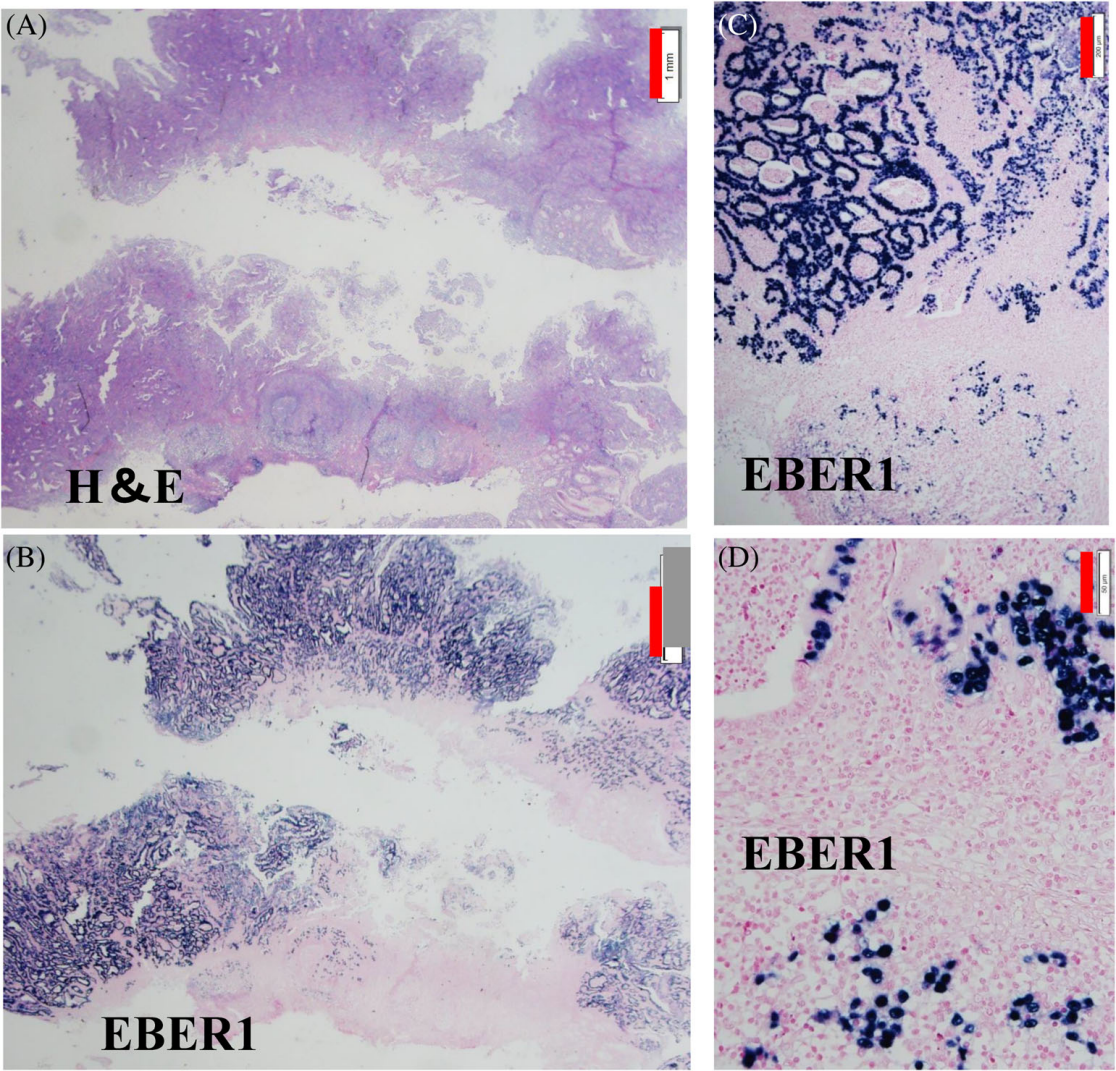
Pathological features of the ESD‐proven early gastric cancer lesion with differentiated and undifferentiated adenocarcinoma mixed‐type histology. The lesion was confirmed to be EBVaGC. (A) Low‐power view of ESD specimen. Hematoxylin and eosin (H&E) staining (×12). ESD reveals mixed‐type histology. The mucosal part consists of differentiated type adenocarcinoma. However, lymphocyte infiltration‐rich undifferentiated type adenocarcinoma part invades the submucosal layer. Scale bar: 1 mm. (B) Low‐power view of ESD specimen. EBER1‐ISH (×12). All cancer cell nuclei were EBER1 positive and the EGC lesion was confirmed to be EBVaGC. Scale bar: 1 mm. EBV‐negative non‐cancer gastric epithelial and immune cells were EBER1 negative. They are internal negative controls in EBER1 ISH. (C) EBER1‐ISH features of the mucosal part of the tumor (×100). All nuclei of the differentiated type tumor are EBER1 positive. Scale bar: 200 μm. (D) EBER1‐ISH features of undifferentiated adenocarcinoma part invading the submucosal layer (×400) Scale bar: 50 μm. All cancer cell nuclei are EBER1 positive. Nuclei of lymphocytes are EBER1 negative.

This EBVaGC lesion was the protruding type (0‐I) EGC, almost three centimeters in diameter and located at the greater curvature of the remnant stomach (Figures 1 and 2). Gastric biopsy classified it into Group 5 (differentiated type) and the tumor was endoscopically limited to within the mucosal layer. The prediction for the mixed‐type ESD result was difficult. However, ESD revealed mixed‐type histology with the undifferentiated CLS part invading the submucosal layer. That patient underwent additional total gastrectomy of the remnant stomach. There was no residual tumor or LN metastasis.

The other 12 ESD‐proven EGC lesions (92.3%) with mixed‐type histology were not CLS and were EBER1‐negative. Eight of the twelve EBER‐1 negative patients underwent additional surgery and six patients had LN metastasis or residual tumors.

## 4 DISCUSSION

In 2014, The Cancer Genome Atlas (TCGA) network characterized gastric cancers into four molecular subtypes: EBV‐positive tumors, microsatellite instable tumors, genomically stable tumors, and tumors with chromosomal instability.^14^ EBVaGC is a specific GC subtype that is host cell genome methylation‐rich with EBV latent infection in all cancer cell nuclei.^14^ It is mainly undifferentiated type adenocarcinoma but with a low risk of LN metastasis in the early stage and with a relatively favorable prognosis in the advanced stage.^15^, ^16^, ^17^ This nature of EBVaGC suggests that the detection of EBV in cancer cells of EGC may have some impact on the therapeutic strategy.

Endoscopically, many cases of EBVaGC mainly have a depressed shape with a submucosal tumor‐like protruding portion. However, differential diagnosis of EBVaGC from other uncommon gastric tumors such as mucinous adenocarcinoma, gastric lymphoma, and endocrine cell carcinoma is not easy.^18^

Ordinarily, a gastric biopsy takes a small specimen from a superficial portion of a GC lesion. When the GC lesion is of mixed‐type histology, a gastric biopsy specimen may represent only a differentiated‐type superficial portion and prediction of mixed type‐histology is difficult.

In contrast, in EBVaGC, EBER1 is positive in cancer cell nuclei even in the superficial differentiated‐type portion. Therefore, EBVaGC is detectable using EBER1‐ISH for superficial gastric biopsy specimens. If EBVaGC has a large part of EGC with mixed‐type histology, EBER1‐ISH for the gastric biopsy specimen may be useful for the prediction of mixed‐type histology. Unfortunately, however, in our present results, EBVaGC was not frequent (7.7%) in EGCs with mixed‐type histology. Thus, prediction of mixed‐type histology using EBER1‐ISH for gastric biopsy specimens seems difficult.

Recently, accumulating data shows that early EBVaGC that invades the submucosal layer without lymphovascular invasion has a low risk of LN metastasis.^19^, ^20^ This may mean that when an ESD‐proven EGC lesion is confirmed as EBVaGC, additional surgical operation may not be needed. Our surgically operated case of EBVaGC had an undifferentiated part that invaded the submucosal layer but no LN metastasis.

In many cases, EBV GC is located in the upper part of the stomach and remnant stomach. Therefore, diagnosis of EBVaGC may support the application of less invasive ESD and may prevent possible excessive surgery such as total gastrectomy.

In the present study, EBVaGC was not frequent in EGCs with mixed‐type histology. Thus, prediction of mixed‐type histology using EBER1‐ISH for gastric biopsy specimens seems difficult. An additional EBV test for ESD‐proven EGC lesions with mixed‐type histology may be useful for decision making on additional treatment.

## AUTHOR CONTRIBUTIONS

**Hideo Yanai:** Conceptualization (lead); project administration (equal); resources (lead); writing – original draft (lead). **Daisuke Chihara:** Data curation (equal); methodology (equal); resources (equal). **Megumi Harano:** Data curation (equal); methodology (equal); resources (equal). **Eiki Sakaguchi:** Data curation (equal); methodology (equal); resources (equal). **Seiji Kaino:** Formal analysis (equal); investigation (equal); supervision (equal). **Tomoyuki Murakami:** Investigation (equal); methodology (equal); writing – review and editing (equal). **Jun Nishikawa:** Formal analysis (equal); investigation (equal); project administration (equal); supervision (equal); writing – review and editing (equal).

## CONFLICT OF INTEREST

The authors have stated explicitly that there are no conflicts of interest in connection with this article.

## ETHICS STATEMENT

This retrospective study was approved by the institutional review board. All procedures performed in studies involving human participants were in accordance with the ethical standards of the institutional research committee and with the 1964 Helsinki declaration and its later amendments. Informed consent was obtained from the patients.
